# Supplementary material for: Identifying dementia using medical data linkage in a longitudinal cohort study: Lothian Birth Cohort 1936
Source: BMC Psychiatry. 2023 May 1;23:303. doi: 10.1186/s12888-023-04797-7 (PMC10152609; doi:10.1186/s12888-023-04797-7)
Supplement: Supplementary file 2 — Additional file 2. Approximate number of person-hours required for each ascertainment phase. [file 12888_2023_4797_MOESM2_ESM.docx]

|  | **Number of researchers involved** | **Approximate number of hours (averaged across researchers)** | **Total** |
| --- | --- | --- | --- |
| **Planning stage** | 4 | 2 | 8 |
| **Phase 1** | 6 | 80 | 400 |
| **Phase 2** | 5 | 4 | 20 |
| **Phase 3** | 9 | 6 | 51 |
|  |  |  |  |
| **TOTAL** |  |  | **489** |

Additional File 2: Approximate number of person-hours required for each ascertainment phase
